# The Relevance of Toxic AGEs (TAGE) Cytotoxicity to NASH Pathogenesis: A Mini-Review

**DOI:** 10.3390/nu11020462

**Published:** 2019-02-22

**Authors:** Akiko Sakasai-Sakai, Takanobu Takata, Jun-ichi Takino, Masayoshi Takeuchi

**Affiliations:** 1 Department of Advanced Medicine, Medical Research Institute, Kanazawa Medical University, 1-1 Daigaku, Uchinada, Kahoku, Ishikawa 920-0293, Japan; takajjjj@kanazawa-med.ac.jp (T.T.); takeuchi@kanazawa-med.ac.jp (M.T.); 2 Department of Biochemistry, Faculty of Pharmaceutical Sciences, Hiroshima International University, 5-1-1, Hirokoshingai, Kure, Hiroshima 737-0112, Japan; j-takino@ps.hirokoku-u.ac.jp

**Keywords:** non-alcoholic fatty liver disease (NAFLD), non-alcoholic steatohepatitis (NASH), advanced glycation end-products (AGEs), glyceraldehyde (GA), glyceraldehyde-derived AGEs, toxic AGEs (TAGE), hepatocytes, hepatocyte stellate cell (HSCs)

## Abstract

Non-alcoholic fatty liver disease (NAFLD) is currently the most common feature of chronic liver disease. Non-alcoholic steatohepatitis (NASH) is a severe form of NAFLD, and one of its risk factors is hyperglycemia. The chronic ingestion of excessive amounts of high-fructose corn syrup is associated with an increased prevalence of fatty liver. Under hyperglycemic conditions, advanced glycation end-products (AGEs) are generated through a non-enzymatic glycation reaction between the ketone or aldehyde groups of sugars and amino groups of proteins. Glyceraldehyde (GA) is a metabolic intermediate of sugars, and GA-derived AGEs (known as toxic AGEs (TAGE)) have been implicated in the development of NASH. TAGE accumulates more in serum or liver tissue in NASH patients than in healthy controls or patients with simple steatosis. Furthermore, the TAGE precursor, GA, causes cell damage through protein dysfunctions by TAGE modifications and induces necrotic-type hepatocyte death. Intracellular TAGE may leak outside of necrotic-type cells. Extracellular TAGE then induce inflammatory or fibrotic responses related to the pathology of NASH in surrounding cells, including hepatocytes and hepatic stellate cells. This review focuses on the contribution of TAGE to the pathology of NASH, particularly hepatic cell death related to NASH.

## 1. Introduction

Non-alcoholic fatty liver disease (NAFLD) is currently the most common feature of chronic liver disease. NAFLD is one of the phenotypes of metabolic syndrome, and its prevalence is increasing worldwide [1]. The spectrum of NAFLD ranges over simple steatosis, steatohepatitis, fibrosis, and cirrhosis. The histopathological diagnosis of steatosis is established by the presence of a macrovesicular fatty change with or without nonspecific inflammation. Non-alcoholic steatohepatitis (NASH) is considered to be the progressive form of steatosis, characterized by the additional presence of cellular ballooning [2,3]. Its pathology often resembles that of alcoholic fatty liver disease; therefore, a diagnosis can be made with the absence of significant alcohol use (an intake of less than 30 g/day for men and 20 g/day for women), negative hepatitis B and C viral markers, the absence of autoimmune hepatitis, non-use of hepatotoxic drugs or other compounds, or rare genetic forms [4]. The pathogenesis of NAFLD, particularly NASH, overlaps with those of lifestyle-related diseases, such as obesity, type 2 diabetes mellitus (T2DM), insulin resistance (IR), and dyslipidemia [5,6,7]. Since NASH has the potential to progress to severe diseases, including cirrhosis and hepatocellular carcinoma, the clarification of its pathology and establishment of therapeutic strategies are urgently required. Agents with the potential to suppress the inappropriate cell death associated with the pathogenesis of NASH may be therapeutic targets. Hepatic apoptosis, autophagic cell death, necroptosis, pyroptosis, and necrosis have been identified in NASH [8,9]. These types of cell death are involved in the pathogenesis of NASH and NASH-induced liver fibrosis; however, the factors responsible for these diseases have not yet been examined in detail. An excessive intake of high-fructose corn syrup (HFCS) or carbohydrates has been associated with the development of NAFLD and NASH [10,11,12]. Under hyperglycemic conditions, advanced glycation end-products (AGEs) are generated through a non-enzymatic glycation reaction. AGEs exist in various forms, depending on the sugars to be reacted. Among various types of AGEs, AGEs derived from glyceraldehyde (GA), which is one of the fructose and glucose metabolic intermediates, are particularly toxic (named toxic AGEs (TAGE)). TAGE have been implicated in NASH, cancer, infertility, dementia, schizophrenia, and cardiovascular diseases [13,14,15,16,17,18,19,20,21,22,23,24,25]. As for NASH, the serum TAGE level was significantly higher in patients with NASH than in those with simple steatosis and healthy controls [13]. The accumulation of TAGE strongly correlates with the pathology of NASH, suggesting the involvement of TAGE in the pathogenesis of this disease. In this review, we discuss the relationship between NASH and AGEs, particularly TAGE.

## 2. Pathway for the Formation of TAGE

**Figure 1:**
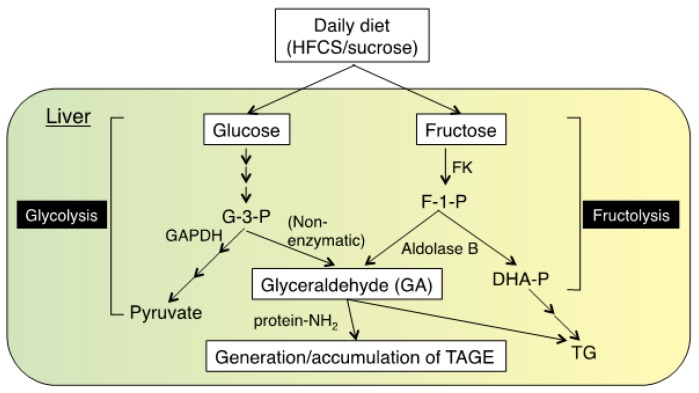
Pathway for the formation of TAGE. Excess consumption of the daily diet (containing HFCS/sucrose) increase glucose/fructose levels. These sugars are metabolized to glyceraldehyde (GA) by glycolysis/fructolysis in the liver. GA reacts with cellular components, including proteins, and generates/promotes the accumulation of TAGE. HFCS: high-fructose corn syrup; FK: fructokinase; GAAPDH: glyceraldehyde-3-phosphate dehydrogenase; G-3-P: glyceraldehyde-3-phosphate; F-1-P: fructose-1-phosphate; DHA-P: dihydroxyacetone-phosphate; protein-NH2: free amino residues of porteins; TAGE: toxic AGEs; TG: triglyceride.

The intake of sugar is needed for the normal function of the body. However, AGEs are formed by the Maillard reaction, a non-enzymatic reaction between reducing sugars (e.g., glucose, fructose, and GA) or carbonyl compounds (e.g., glyoxal, methylglyoxal, 3-deoxyglucosone, and acetaldehyde) and the ε-amino group of lysine residues, guanidino group of arginine residues, or N-terminal α-amino group of proteins. This reaction has been investigated as a phenomenon related to taste and flavor in the field of food science, and is known to occur ubiquitously, even in tissue and blood, under natural conditions. The formation of AGEs and Amadori products, the early stage of glycation (e.g., hemoglobin A1c (HbA1c)), in diabetic patients with high blood glucose levels is greater than that in healthy subjects. HbA1c has clinical usefulness as a marker for blood sugar control in diabetic mellitus. Some AGEs exert toxic effects in mammals, including humans. Several types of aldehydes and carbonyl compounds, including GA, glycolaldehyde, acetaldehyde, glyoxal, methylglyoxal, and 3-deoxyglucosone, quickly form AGEs. Among the different types of AGEs, GA-derived AGEs exhibit strong cytotoxicity and strongly support the concept of TAGE [14,15]. The pathway for the formation of TAGE is as follows: (1) the glycolytic pathway is an important pathway for glucose metabolism (Figure 1, left pathway). An intermediate of this pathway, glyceraldehyde-3-phosphate (G-3-P), is metabolized by glyceraldehyde-3-phosphate dehydrogenase (GAPDH). In the case of decreased GAPDH enzymatic activity, accumulated G-3-P is shifted to another metabolic route, resulting in the accumulation of GA. (2) The fructolysis pathway is another important pathway, particularly in the liver (Figure 1, right pathway). Fructose is phosphorylated by fructokinase (FK) and converted to fructose-1-phosphate (F-1-P). F-1-P is cleaved by aldolase B, and then dihydroxyacetone phosphate and GA is produced. GA is generated by these pathways and promotes the generation/accumulation of TAGE.

## 3. NASH Clinical Specimens and TAGE

TAGE is formed intracellularly and is predicted to spread throughout the body. A previous study reported that serum TAGE levels were higher in T2DM patients than in control subjects [26]. On the other hand, the administration of acarbose, an α-glucosidase inhibitor, reduced postprandial hyperglycemia and TAGE levels in the serum of T2DM patients [27]. These findings suggest that TAGE actually accumulate in serum under hyperglycemic conditions and fluctuate with changes in blood sugar levels. Furthermore, TAGE in serum and tissues correlate with the onset and progression of various diseases, including the chronic complications associated with diabetes mellitus and NASH [13,14,15,16,17,18,19,20,21,22,23,24,25]. Recently, it was reported that fructose is mainly metabolized in the small intestine, but in the case of excess fructose, it is metabolized in the liver [28]. NASH is frequently associated with abnormal glucose/fructose metabolism [9,10,11,12]. Moreover, AGEs generated by the excessive intake of sugar have been implicated in NASH and the development of NAFLD. TAGE have been shown to accumulate in the liver tissues of NASH patients, but to a lesser extent in those with simple steatosis [13]. The accumulation of TAGE, not only in tissue, but also in serum, was previously shown to be greater in NASH patients (9.78 ± 3.73 U/mL) than in healthy controls (6.96 ± 2.36 U/mL) and patients with simple steatosis (7.17 ± 2.28 U/mL) [13]. The measurement of TAGE appeared to able to discriminate NASH from simple steatosis, as shown by the area under the ROC curve of 78% [13]. Furthermore, we found that serum TAGE levels were higher in patients with non-B non-C hepatocellular carcinoma (HCC) than in those with NASH alone [16]. On the other hand, when atorvastatin was administered to NASH patients with lipid metabolism abnormalities, liver function significantly improved with decreases in serum TAGE levels after 6 months of treatment [29]. These findings suggest that the amount of TAGE fluctuates with the pathological condition of NASH.

## 4. Cytotoxicity of TAGE

In NASH, necroinflammatory processes with hepatocyte ballooning, lipoapoptosis, and progressive fibrosis are promoted. These factors potentially contribute to its progression to cirrhosis. Although the suppression of inflammation and fibrosis has potential in the treatment of NASH, the mechanisms underlying NASH-related cell death have not yet been elucidated in detail. Hepatic stellate cells (HSCs), the main extracellular matrix-producing cells, are a key player in fibrosis. Hepatocyte death is one of the central phenomena in the development of NAFLD. In this section, we focus on the relationship between these cell deaths and TAGE.

### 4.1. HSCs

**Figure 2:**
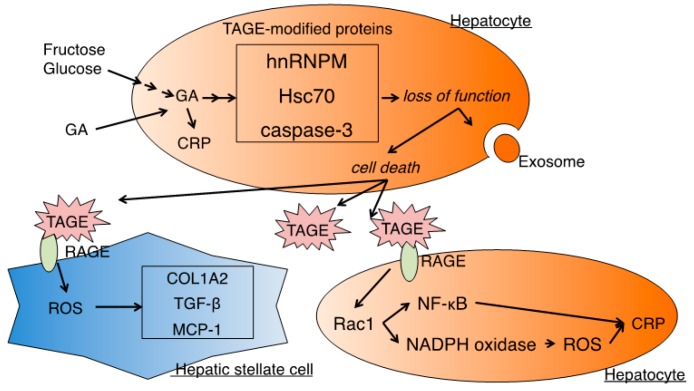
Cytotoxicity of TAGE in hepatocytes and hepatic stellate cells. The accumulation of GA in hepatocytes results in TAGE modifications to cellular components, including proteins. TAGE-modified proteins lose their function, and this ultimately results in hepatocyte death. Hepatocyte death may cause TAGE-modified protein leakage, and extracellular TAGE influence surrounding cells via the TAGE-RAGE axis. GA: glyceraldehyde; CRP: C-reactive protein; TAGE: toxic AGEs; hnRNPM: heterogenous nuclear ribonucleoprotein M; Hsc70: heat shock cognate 70; RAGE: receptor for AGEs; ROS: reactive oxygen species; COL1A2: collagen-type Iα 2; TGF-β: transforming growth factor-β; MCP-1: monocyte chemoattractant protein-1; Rac1: Rs-related C3 botulinum toxin substrate 1; NF-κB: nuclear factor kappa B; NADPH: nicotinamide adenine dinucleotide phosphate.

In addition to the finding of increased TAGE levels in patients with NASH, the mechanisms responsible for liver cell damage have also been elucidated. As NASH progresses, HSCs are active and involved in liver fibrosis. The effects of TAGE on HSCs mediated by the receptor for AGEs (RAGE) on cellular membranes have mainly been studied. A TAGE-modified bovine serum albumin (BSA) treatment of the human HSC line LI90 induced intracellular oxidative stress via RAGE. Furthermore, the mRNA levels of fibrotic genes (α-smooth muscle actin, collagen type lα2, and transforming growth factor-β) were increased by the TAGE-modified BSA treatment of LI90. The expression levels of monocyte chemoattractant protein-1, which is involved in inflammation, were also increased [30]. Therefore, TAGE appear to contribute to the development and progression of hepatic fibrosis through the production of reactive oxygen species and activation of HSCs via RAGE (Figure 2).

### 4.2. Hepatocytes

Hepatocyte dysfunctions play a central role in the progression of NASH in animals and humans. The hepatocyte dysfunction associated with NASH may be attributed to the cytotoxicity of TAGE. TAGE-modified proteins were detected in the human HCC cell lines Hep3B or HepG2 when cells were incubated with high fructose or GA [31,32,33,34]. Furthermore, hepatocyte damage was associated with the accumulation of intracellular TAGE. The treatment of Hep3B or HepG2 cells with GA induced intracellular TAGE accumulation and caused cell death [33,34]. AGEs are known to form intra- and inter-protein crosslink structures [35]. The structural alterations caused by crosslinking in TAGE-modified proteins may result in the loss of appropriate functions. Previous studies reported that the TAGE modifications that impaired the protein functions important for cell maintenance and survival were associated with hepatocyte cytotoxicity (Figure 2). (1) Heterogeneous nuclear ribonucleoprotein M (hnRNPM) was identified as a TAGE-modified protein in Hep3B cells incubated with high fructose or GA-containing media [31,32]. hnRNPM is an RNA-binding protein that contributes to multiple aspects of nucleic acid metabolism, including alternative splicing, mRNA stabilization, and transcriptional and translational regulation. The expression of genes involved in the extracellular space containing extracellular exosomes was shown to be up-regulated by the knockdown of hnRNPM [32]. Although the role of hnRNPM in the extracellular space has remained unclear, genes associated with the extracellular space have potential as biomarkers for NASH [32]. (2) Heat shock cognate 70 (Hsc70) was also found to be modified as TAGE in Hep3B cells following a treatment with GA [33]. Hsc70 widely maintains intracellular protein homeostasis by regulating protein folding, transport, and degradation [36]. Since TAGE-modified Hsc70 lost its chaperone activity in vitro, TAGE-modified Hsc70 did not exhibit chaperone activity in hepatocytes and causes cell death [33]. (3) Caspase-3, which plays a central role in apoptosis, is also a target of TAGE modifications in HepG2 cells treated with GA. A previous study demonstrated that caspase-3 was cleaved and activated to exhibit protease activity during apoptosis [37]. However, increases in TAGE-modified caspase-3 were associated with the loss of enzymatic activity, and modifications by TAGE also suppressed the cleavage of poly (ADP-ribose) polymerase, which is downstream of caspase-3 in the apoptotic cascade. Furthermore, necrotic-type cell death increased with the appearance of TAGE-modified caspase-3 [34]. In the pathology of NASH, various types of hepatocyte death, including apoptosis and necrosis, occur in liver tissue [8,9]. The development of NASH may be associated with increases in intracellular GA and TAGE-modified proteins, and TAGE modifications to proteins, including caspase-3, have been suggested to induce necrotic-type cell death [34]. The leakage of TAGE-modified proteins may also be responsible for increases in TAGE levels in the serum of NASH patients. 

Thus, the accumulation of intracellular TAGE exerts cytotoxic effects on hepatocytes. Extracellular TAGE also promote inflammatory effects in hepatocytes via the TAGE-RAGE interaction, which is also characteristic of the pathology of NASH. The expression of the inflammatory marker C-reactive protein (CRP) was up-regulated by the treatment of the RAGE-expressing HCC cell line Hep3B with TAGE-modified BSA [38,39]. CRP expression was mediated by Ras-related C3 botulinum toxin substrate 1, and followed by the activation of nuclear factor kappa B or nicotinamide adenine dinucleotide phosphate (NADPH) oxidase, which are strongly expressed in NASH patients [40,41]. NADPH oxidase generates reactive oxygen species, which enhance inflammation in Hep3B cells (Figure 2) [38]. In addition to the effects of extracellular TAGE, the accumulation of intracellular TAGE exerts inflammatory effects in hepatocytes. A previous study demonstrated that a treatment with GA caused the accumulation of TAGE in Hep3B cells and increased CRP expression levels [33]. Increases in CRP levels were suppressed to control levels by a pretreatment with aminoguanidine, an inhibitor of the formation of AGE, suggesting that the formation and accumulation of intracellular TAGE induce inflammatory responses [33]. These findings indicate that TAGE induce inflammation via their intracellular and extracellular effects and contribute to the onset and progression of NASH (Figure 2).

## 5. Conclusions

The multiple parallel hit hypothesis has been proposed as a mechanism for the onset of NASH [42]. ‘Multiple’ includes IR, nutrient factors, host heritability, and intestinal microflora. In addition, since NAFLD and NASH are frequently associated with the abnormal metabolism of glucose, the production of AGEs, particularly TAGE, in hyperglycemia is considered to contribute to the onset of these diseases. NASH may progress to liver cirrhosis and liver cancer, and it currently has no effective therapeutic agent. Hence, a clearer understanding of the mechanisms responsible for the development of NASH may contribute to the development of effective therapies. HSCs and hepatocytes are important cells in the progression of NASH and are strongly affected by TAGE [31,32,33,34]. Once GA forms in cells, proteins that function as chaperones and/or in apoptosis are modified by TAGE and lose their function [33,34]. The accumulation of TAGE has been correlated with necrotic-type cell death in HepG2 cells [34]. Since necrotic-type cell death is associated with the leakage of intracellular constituents, necrotic-type HepG2 cell death caused by the accumulation of TAGE may result in the leakage of intracellular components, including TAGE, into the extracellular space. Extracellular TAGE have been shown to induce inflammatory responses in hepatocytes harboring RAGE on their surface [38,39]. Furthermore, in HSCs, increases in oxidative stress and fibrosis were found to be accelerated by extracellular TAGE, which reflects the fibrosis characteristic of the NASH pathology [30]. Therefore, TAGE leakage from necrotic-type hepatocytes may cause fibrosis and inflammation in surrounding HSCs and hepatocytes, respectively. These findings provide novel insights into cell death associated with NASH and the development of therapeutic anti-inflammation targets for its treatment. TAGE that leak from hepatocytes may escape into the blood and circulate throughout the body. The pathology of NASH correlates with serum TAGE levels, and the accumulation of TAGE has been shown to increase with the progression of the disease state [13,14,15,16]. Although various factors have been examined as markers for the diagnosis of NASH, an effective marker has not yet been identified. We propose that TAGE is not only a factor responsible for the pathology of NASH, but also has a potential as a marker for its diagnosis.
